# U. S. V. M. A. Appointments

**Published:** 1896-10

**Authors:** 


### U. S. V. M. A. APPOINTMENTS.

PRESIDENT OSGOOD announces the following appointments for the ensuing year.

Army Legislation: J. P. Turner, Chairman, Ft. Meyer, Va.; John R. Hart, 2577 Amber St., Philadelphia, Pa.; Austin Peters, 40 Water St., Boston, Mass.

Finance Committee: W. Horace Hoskins, Chairman, 3452 Ludlow St., Philadelphia; James B. Rayner, West Chester, Pa.; S. Stewart, 7½ S. James St., Kansas City, Kan.

Committee for Preparation of Resolutions to be presented to the Local Committee of Arrangements of Buffalo Meeting: Leonard Pearson, Chairman, Harrisburg, Pa.; W. Horace Hoskins, Philadelphia; F. H. Osgood, 50 Village St., Boston; S. Stewart, Kansas City, Kan.

Publication Committee: W. L. Williams, Chairman, Ithaca, N. Y.; Nelson P. Hinkley, Buffalo, N. Y.; Sesco Stewart, Kansas City, Kan.

